# A difficult-to-diagnose fibromatosis-like metaplastic carcinoma of the breast: a case report

**DOI:** 10.1186/s40792-021-01110-0

**Published:** 2021-01-12

**Authors:** Daiki Takatsuka, Hiroyuki Ogura, Yuko Asano, Akiko Nakamura, Kei Koizumi, Norihiko Shiiya, Satoshi Baba

**Affiliations:** 1 First Department of Surgery, Hamamatsu University School of Medicine, 1-20-1 Handayama, Higashi-ku, Hamamatsu, Shizuoka 431-3192 Japan; 2 Department of Pathology, Hamamatsu University Hospital, 1-20-1 Handayama, Higashi-ku, Hamamatsu, Shizuoka 431-3192 Japan

**Keywords:** Breast cancer, Metaplastic carcinoma, Fibromatosis-like metaplastic carcinoma

## Abstract

**Background:**

Fibromatosis-like metaplastic carcinoma (FLMCa), classified as a metaplastic carcinoma of the breast, is a very rare type of metaplastic carcinoma. We report a case of FLMCa that was difficult to diagnose.

**Case presentation:**

The patient was a 56-year-old postmenopausal woman who presented with a left-sided breast mass. A 1.3-cm irregular mass was found in the lower outer quadrant of the left breast on breast ultrasonography. She underwent core needle biopsy and vacuum-assisted biopsy, but the pathological findings only revealed inflammatory cell infiltration and a high level of fibrosis, with no malignant findings. At 3 months follow-up, she underwent a repeat breast ultrasonography, which revealed an increase in the size of the mass to 1.8 cm, and a repeat core needle biopsy, which showed a few spindle cells and squamous cells positive for cytokeratin (CK)5/6 and AE1/AE3, leading to the suspicion of FLMCa. Since the amount of tissue was insufficient to establish a definitive diagnosis, she underwent a lumpectomy. We found low-grade and slightly atypical spindle cells and partly atypical spindle cell carcinoma and squamous cell carcinoma. CK5/6 and α-SMA were positive, thus confirming FLMCa. Because the margins on the edge of the nipple side and anterior side were “ink on tumor”, she underwent a mastectomy and sentinel lymph node biopsy. After the surgery, she received adjuvant chemotherapy. At 3 years and 8 months of follow-up, no recurrent or metastatic lesions were identified in her body.

**Conclusions:**

FLMCa should be considered in the differential diagnosis when collagenous fibers are proliferating and malignancy is clinically suspected. Immunohistochemical analysis may be helpful in confirming this diagnosis.

## Background

Fibromatosis-like metaplastic carcinoma (FLMCa) was proposed by Gobbi et al. in 1999 [1], and classified as a metaplastic carcinoma in the fifth edition of the World Health Organization (WHO) classification of breast tumors in 2019 [2]. Metaplastic carcinoma of the breast is a relatively rare histological type, accounting for 0.2–1% of all invasive breast cancers [2], and FLMCa has been reported even less frequently. It is very difficult to diagnose FLMCa accurately because its histopathological findings are similar to those of benign tumors, such as fibromatosis and nodular fasciitis [1]. We report a case of FLMCa that was difficult to diagnose based on tissue biopsy.

## Case presentation

A 56-year-old postmenopausal woman presented with a left-sided breast mass for which she consulted a nearby physician. She underwent mammography and breast ultrasonography and was referred to our department for suspected breast cancer.

She harbored a 2-cm well-defined and mobile elastic hard mass in the lower outer quadrant of the left breast. The axillary lymph nodes were not palpable.

**Fig. 1:**
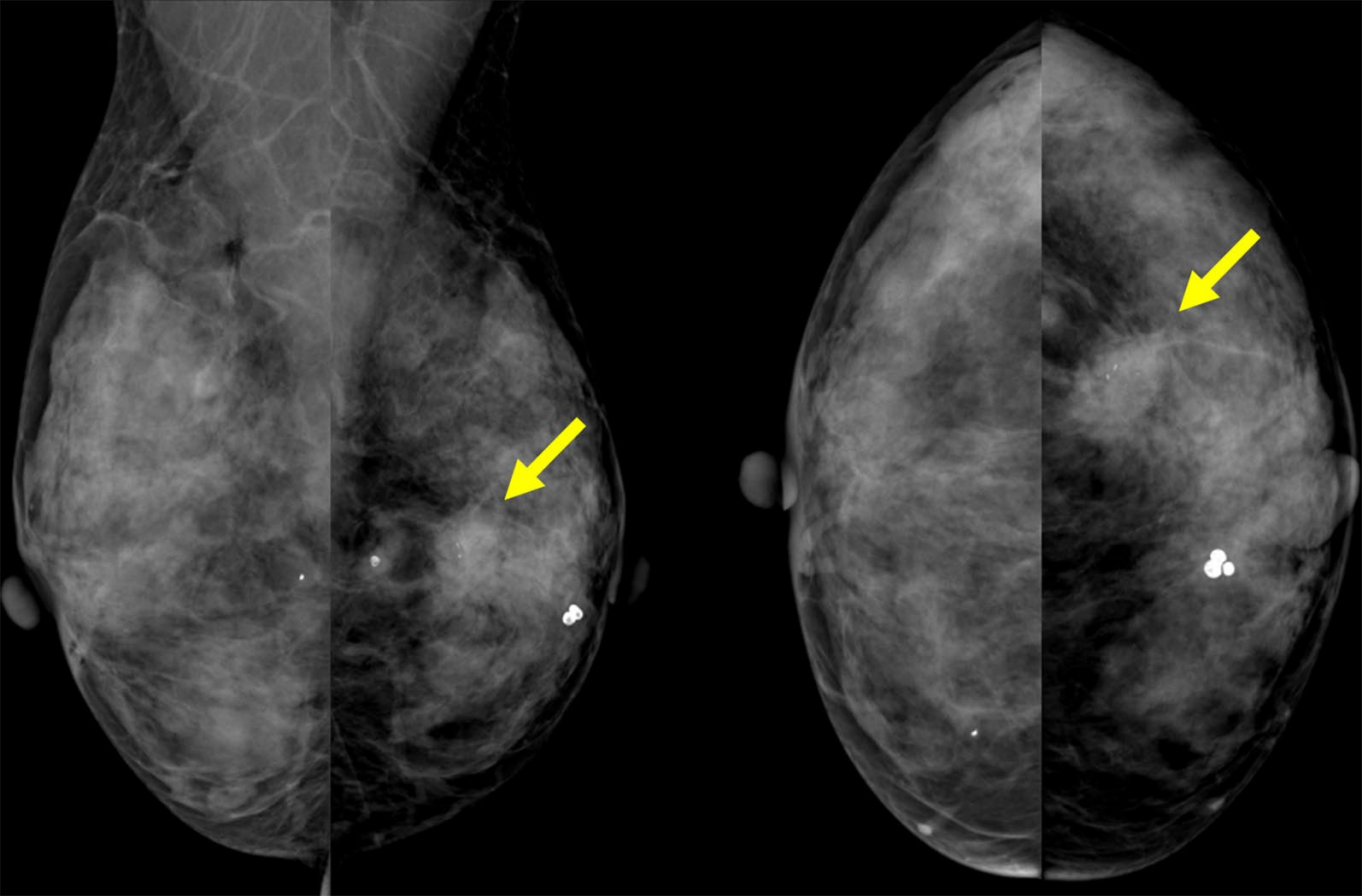
Mammography. In the lower and outer areas of the left breast, a 2-cm micro-lobulated high-density mass was observed (arrows)

In the middle and outer areas of the left breast, a 2-cm micro-lobulated high-density mass was observed on mammography (Fig. 1). Polymorphic calcifications were found within the tumor.

**Fig. 2:**
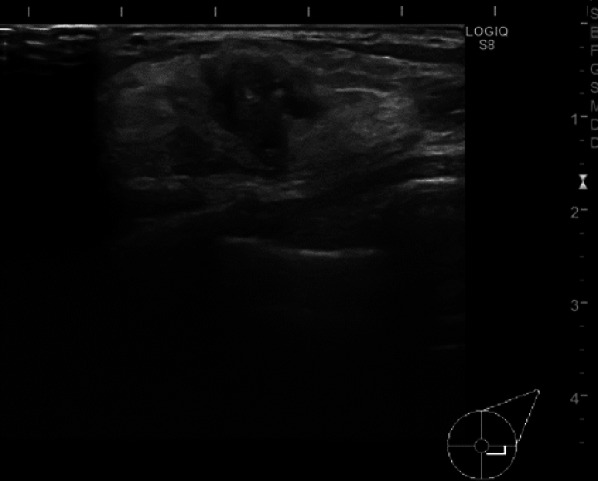
Breast ultrasonography. A 1.3 × 1.0 × 1.1-cm irregular hypoechoic mass with angular margin mass was found in the lower outer quadrant of the left breast

A 1.3 × 1.0 × 1.1-cm irregular hypoechoic mass with angular margin was found in the lower outer quadrant of the left breast on breast ultrasonography (Fig. 2). Many calcifications were observed within the tumor.

**Fig. 3:**
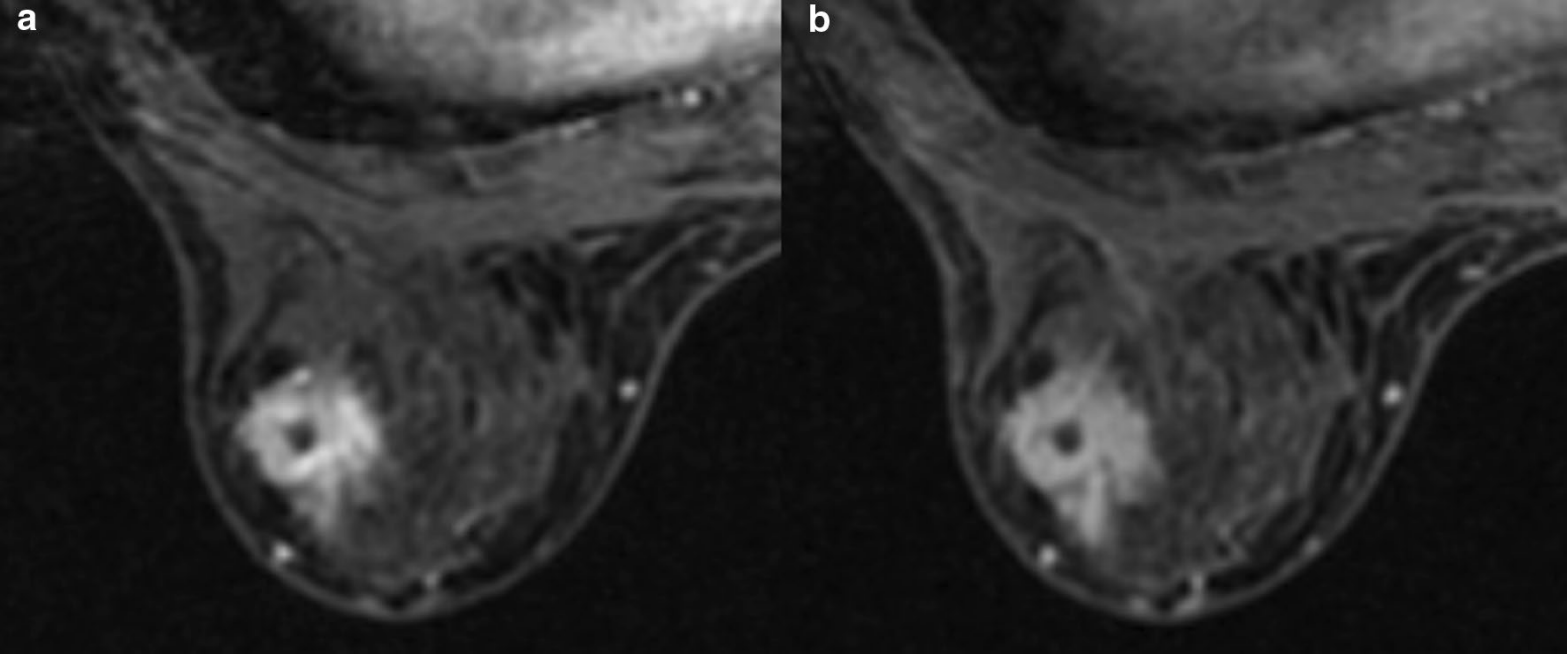
Magnetic resonance imaging. **a** A 1.9 × 1.8 × 1.7-cm irregular tumor was observed in the lower, outer quadrant of the left breast, which was well imaged in the early phase. **b** The tumor was washed out in the late phase

A 1.9 × 1.8 × 1.7-cm irregular tumor was observed in the lower outer quadrant of the left breast, which was well imaged in the early phase and washed out in the late phase on magnetic resonance imaging (Fig. 3a, b). The internal enhancement characteristics were heterogeneous.

These tests showed that the tumor was likely malignant; therefore, she underwent core needle biopsy (16G). Fibrosis was observed in 4 of 4 specimens, and duct components and squamous epithelium-like cells were partially observed in 2 specimens. Spindle cells were also observed, but the mitosis rate and nuclear grade were very low, and AE1/AE3 were immunohistologically negative. It was very difficult to ascertain whether the lesions were reactive or neoplastic.

Because these imaging findings strongly suggested that the tumor was malignant, she underwent vacuum-assisted biopsy (10G). However, the pathological findings only revealed inflammatory cell infiltration and a high level of fibrosis. Fibrosis was observed in 6 of 7 specimens, and duct components were partially observed in 2 specimens; however, there were no malignant findings; therefore, we decided to conduct careful follow-up.

After 3 months, she underwent a repeat breast ultrasonography, which revealed that the mass had grown to 1.8 × 1.7 × 1.5 cm. She underwent a repeat core needle biopsy (16G).

**Fig. 4:**
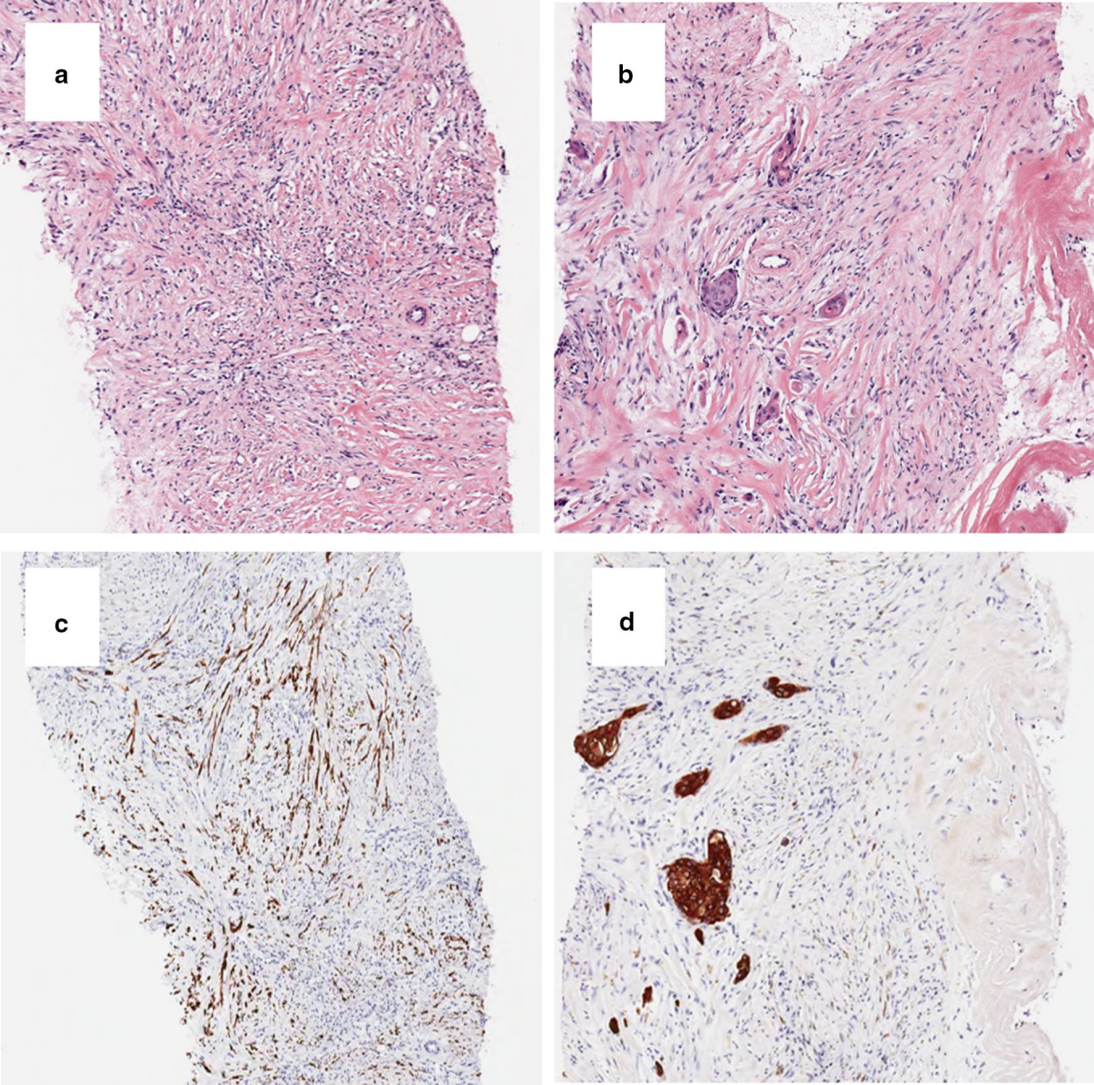
Immunohistological findings of the biopsy. **a** Proliferation of fibroblast-like spindle cells was observed (H&E × 100). **b** Partial agglomeration of squamous cell-like cells with low-grade atypical nuclei was observed (H&E × 100). **c** CK5/6 were positive in spindle cells (× 100). **d** AE1/AE3 were positive in squamous cells (arrows × 100). *H&E* hematoxylin and eosin, *CK* cytokeratin

Proliferation of fibroblast-like spindle cells and partial agglomeration of squamous-like cells with low-grade atypical nuclei were observed (Fig. 4a, b). These findings were observed in 6 of 6 specimens.

Immunohistochemistry (IHC) antibody markers (cytokeratin (CK)5/6 and AE1/AE3) were positive in spindle cells and squamous cells (Fig. 4c, d). The other antibody markers (CK14, p63, and p40) were also positive in these cells. CAM5.2 was partially positive in the squamous cells, but negative in the spindle cells.

Spindle cells were mainly proliferating, and epithelial components were partially aggregated; therefore, we suspected that this tumor was FLMCa, but the amount of tissue was insufficient to establish a definitive diagnosis. To confirm the diagnosis, lumpectomy with a 1.0-cm margin was performed.

**Fig. 5:**
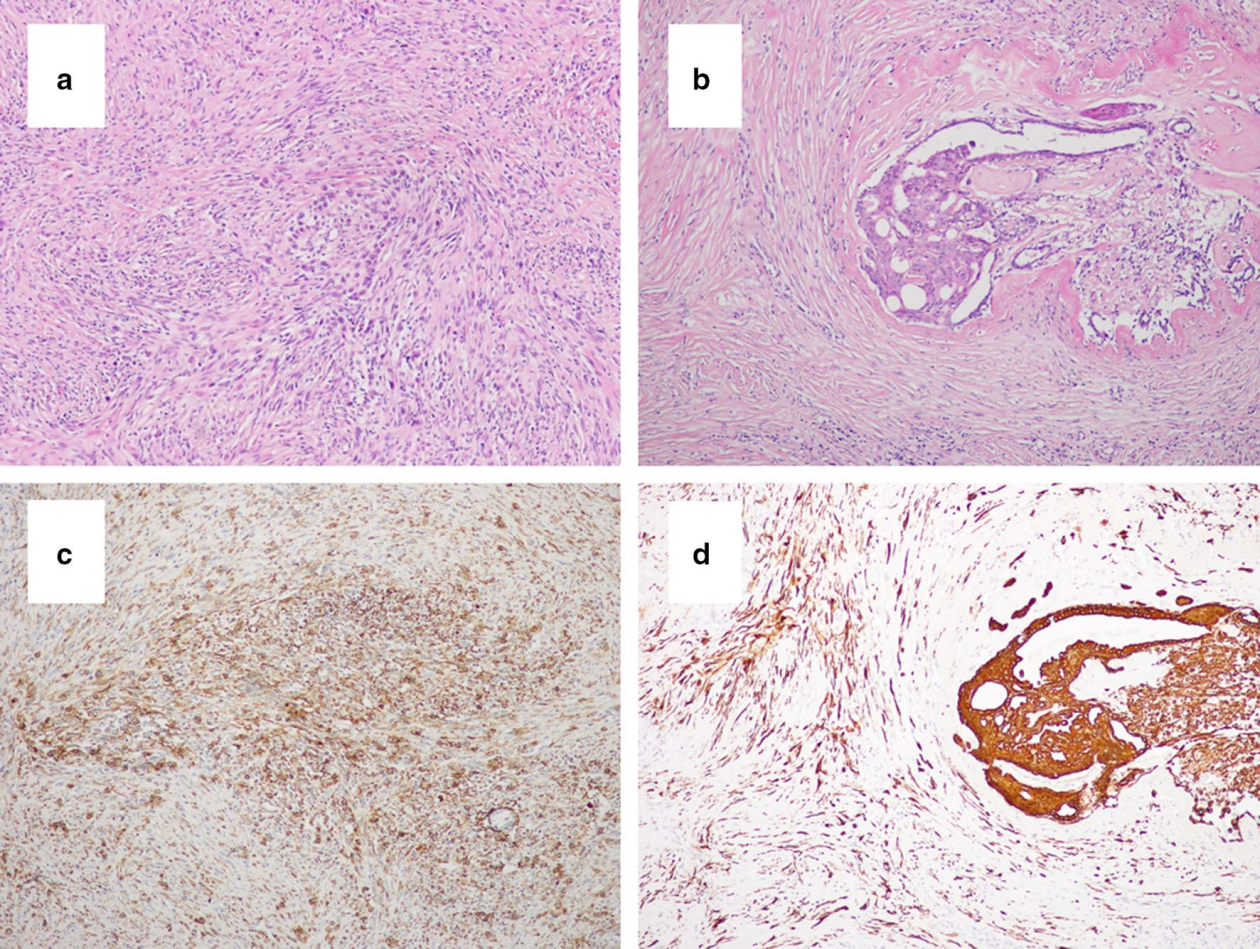
Immunohistological findings of the lumpectomy specimen. **a** Low-grade and slightly atypical spindle cells and partly atypical spindle cell carcinoma were observed (H&E × 100). **b** Partly atypical squamous cell carcinoma was observed (H&E × 100). **c** α-SMA was positive in spindle cells. **d** CK5/6 were positive in squamous cells. *H&E* hematoxylin and eosin, *SMA* smooth muscle actin, *CK* cytokeratin

The cut surface of the lumpectomy specimen revealed a firm, white, nodular tumor measuring 2.0 × 2.0 × 1.5 cm with infiltrative borders. We found low-grade and slightly atypical spindle cells and partly atypical spindle cell carcinoma and squamous cell carcinoma (Fig. 5a, b). IHC antibody markers (α-SMA and CK5/6) were positive in spindle cells and squamous cells (Fig. 5c, d). The other antibody markers (CK14, p63, and p40) were also positive in these cells. Based on these pathological findings, we diagnosed FLMCa. The tumor invasive size was 2.3 cm (pT2). The tumor cells were negative for estrogen receptor (ER), progesterone receptor (PgR), and HER2. The Ki-67 labeling index was 12% in the tumor cells. The margins on the edge of the nipple side and anterior side were “ink on tumor”. She then underwent a mastectomy and sentinel lymph node biopsy. No invasive lesions were present, but lobular carcinoma in situ was widely present in the surgical specimen. The sentinel lymph node biopsy results were negative.

After the surgery, she received adjuvant chemotherapy (docetaxel + cyclophosphamide; TC). In the second cycle, she developed allergic symptoms. Therefore, she could not continue the TC, and received 3 cycles of another type of chemotherapy (epirubicin + cyclophosphamide; EC). At 3 years and 8 months of follow-up, no recurrent or metastatic lesions were identified in her body.

## Conclusions

Gobbi et al. selected 241 cases of metaplastic features in breast tumors and investigated 30 cases in which low-grade spindle cells accounted for more than 95% of the total area of the tumor and less than 5% were epithelial cells or low-grade carcinoma [1]. Although the local recurrence rate was high, no lymph node recurrence or distant metastasis was observed; therefore, they did not use the terminology “carcinoma” and proposed the concept of “metaplastic breast tumor with a dominant fibromatosis-like phenotype”. Local recurrences occurred in 7 of 8 cases involving excision and 1 of 10 cases involving wide excision, but none of them involved distant metastases despite no drug therapy. No lymph node metastasis was observed in 10 patients who underwent axillary lymph node dissection.

Sneige et al. also investigated 24 cases with the same definition, but found local recurrence in 2 cases and lung metastasis in 2 cases [3]. Therefore, they used the terminology “low-grade (fibromatosis-like) spindle cell carcinoma”. Later, Lamovec et al. reported a case of lymph node metastasis [4]. Based on these reports, FLMCa is classified as a special type of metaplastic carcinoma in the WHO classification of breast tumors, fifth edition (2019).

Histopathologically, the lesions are characterized by the proliferation of largely spindle/fibroblastic or stellate/myofibroblastic cell components of varying cellularity, which are intermixed with various amounts of collagenous stroma [3]. The majority of tumors have mild chronic inflammatory infiltrates admixed with the tumor cells. Focal squamous differentiation was observed. Tumor cells have a pale acidophilic cytoplasm with round-to-oval nuclei and occasional small nucleoli. The tumor cells are of low nuclear grade, and mitotic activity is also very low.

Immunohistologically, AE1/AE3, CAM5.2, CK, vimentin, and α-SMA are often positive in FLMCa, especially AE1/AE3 is highly positive [1, 3]. The other antibodies, ER, PgR, and HER2, are often negative, similar to that in normal metaplastic carcinoma.

The differential diagnoses considered are nodular fasciitis, inflammatory myofibroblastic tumors, pseudoangiomatous stromal hyperplasia, myofibroblastoma, and fibromatosis [1, 3]. The reports by Gobbi et al. and Sneige et al. suggested that approximately one-third of the initial histological diagnoses are suspected to be fibromatosis. When a tumor has large amounts of fibrous components such as fibromatosis, it is necessary to consider FLMCa and do immunohistochemical studies (CK and AE1/AE3).

In this case, we were initially unable to diagnose the patient with FLMCa because the first and second biopsies revealed few tumor cells that were slightly atypical and were negative for AE1/AE3. This caused us to follow-up on her.

Inadequate excision could be attributed to local recurrence [1]; therefore, we decided to perform additional resection because the margins on the edge of the nipple side and anterior side were “ink on tumor” in the first surgery. We could not redo a lumpectomy for cosmetic reasons; therefore, we performed a mastectomy.

There were few lymph node metastases; however, Lamovec et al. reported a case of lymph node metastasis [4]. We also performed sentinel lymph node biopsy, similar to the standard surgery for invasive carcinoma.

Pathological findings in the adjacent breast parenchyma typically include radial scars, complex sclerosing lesions, intraductal papillomas, low-grade ductal carcinoma in situ, and lobular carcinoma in situ [1, 3]. In this case, lobular carcinoma in situ was widely present in the surgical specimen.

Previous reports indicated that many patients did not receive chemotherapy [1, 3], but few relapses occurred. However, a few cases of distant metastasis have been reported [3]. The benefit of chemotherapy is controversial because there were few reports of FLMCa, and previous reports have reported that the median follow-up periods were 27 months (range, 6–88 months) and 33 months (range, 8–90 months), respectively, which were very short [1, 3]. Therefore, we suggest that our patient receive standard adjuvant chemotherapy. At 3 years and 8 months of follow-up, she had no recurrent or metastatic lesions. Whether chemotherapy is effective is unknown, and more cases are needed.

In conclusion, it is very difficult to diagnose FLMCa accurately because the histopathological findings are similar to those of benign tumors, such as reactive fibrosis and benign sclerosing lesions. When collagenous fibers are proliferative and malignancy is clinically suspected, such as in this case, FLMCa should be considered in the differential diagnosis. Immunohistochemical analysis may be helpful in making this diagnosis. Since local recurrence occurs at a high rate that is only found by excision, surgical procedures with sufficient margins are required.

## Data Availability

All data generated or analyzed during this study are included in this published article [and its supplementary information files].

## Abbreviations

CK: Cytokeratin
ER: Estrogen receptor
FLMCa: Fibromatosis-like metaplastic carcinoma
HER2: Human epidermal growth factor receptor 2
IHC: Immunohistochemistry
PgR: Progesterone receptor
SMA: Smooth muscle actin
WHO: World Health Organization
